# Roles for the Endoplasmic Reticulum in Regulation of Neuronal Calcium Homeostasis

**DOI:** 10.3390/cells8101232

**Published:** 2019-10-10

**Authors:** Nicholas E. Karagas, Kartik Venkatachalam

**Affiliations:** 1 Department of Integrative Biology and Pharmacology, McGovern Medical School at the University of Texas Health Sciences Center (UTHealth), Houston, TX 77030, USA; Nicholas.E.Karagas@uth.tmc.edu; 2 Graduate Program in Biochemistry and Cell Biology, MD Anderson Cancer Center and UTHealth Graduate School of Biomedical Sciences, Houston, TX 77030, USA

**Keywords:** neuronal calcium signaling, endoplasmic reticulum, bioenergetics, neurodegeneration, IP_3_R, ryanodine receptor, neurotransmission, synaptic transmission

## Abstract

By influencing Ca^2+^ homeostasis in spatially and architecturally distinct neuronal compartments, the endoplasmic reticulum (ER) illustrates the notion that form and function are intimately related. The contribution of ER to neuronal Ca^2+^ homeostasis is attributed to the organelle being the largest reservoir of intracellular Ca^2+^ and having a high density of Ca^2+^ channels and transporters. As such, ER Ca^2+^ has incontrovertible roles in the regulation of axodendritic growth and morphology, synaptic vesicle release, and neural activity dependent gene expression, synaptic plasticity, and mitochondrial bioenergetics. Not surprisingly, many neurological diseases arise from ER Ca^2+^ dyshomeostasis, either directly due to alterations in ER resident proteins, or indirectly via processes that are coupled to the regulators of ER Ca^2+^ dynamics. In this review, we describe the mechanisms involved in the establishment of ER Ca^2+^ homeostasis in neurons. We elaborate upon how changes in the spatiotemporal dynamics of Ca^2+^ exchange between the ER and other organelles sculpt neuronal function and provide examples that demonstrate the involvement of ER Ca^2+^ dyshomeostasis in a range of neurological and neurodegenerative diseases.

## 1. Overview

For well over a century, perhaps starting with the remarkable drawings of Ramón y Cajal [1], neurons have been appreciated for their exquisite structural intricacy. Mirroring the complexity of neuronal morphology, architecture of their organelles varies significantly from one subcellular location to the next. For instance, the endoplasmic reticulum (ER), whose surface area vastly exceeds that of the plasma membrane [2], is comprised of tightly-packed interconnected sheets in cell bodies and dendrites, whereas the same organelle in axons takes on a characteristically tubular shape [3,4,5,6,7]. Recent studies have suggested that the morphological features of the ER are not ‘accidental’ consequences of neuronal arborization. Rather, the observed structural heterogeneity is inherent to the functional diversity and pleiotropy of these organelles in different neuronal compartments. In the following sections, we describe our current understanding of the mechanisms involved in the regulation of ER Ca^2+^ homeostasis through the lens of neuronal architecture and elaborate upon how changes in ER Ca^2+^ dynamics across space and time sculpt various aspects of neuronal function.

## 2. Neuronal Polarity Imposes Structural Constraints on the ER 

Most neurons exhibit stereotypical compartmentalization that arises from partitioning into functional modules called dendrites, cell bodies, and axons. Whereas cell bodies house the nucleus and serve as de facto guides of overall cellular function, dendrites and axons are responsible for the transmission of electrical and chemical signals across neuronal networks. Dendrites, the signal receiving compartments, are comprised of tiny protrusions called spines, each of which contains a specialized ER structure called the spine apparatus [5,6]. The spine apparatus has roles in the regulation of spine morphogenesis, Ca^2+^ dynamics, and synaptic plasticity [5,8,9]. Structurally, the spine apparatus is made up of interconnected stacks of ER, and typically occupies <5% of the spine volume [10]. Despite the relatively small fraction of the total spine volume, the complexity of ER structure, in terms of branching and local arborization, increases dramatically as the ER traverses into spines from the ‘bulk’ dendrite [11]. This increase in structural complexity serves to limit the movement of proteins embedded in the ER, thus facilitating the translocation of proteins from the spine apparatus to local plasma membrane [11,12]. These findings underscore the validity of the notion that the structural heterogeneity of the ER is not simply a passive outcome of neuronal arborization, but rather represents an example of ‘function arising from form’. 

The morphology of ER in neuronal cell bodies is similar to that observed in spines, and resembles stacked sheets connected by helicoidal structures interspersed between the sheets [4]. ER sheets in neuronal soma and dendrites exhibit continuity and dynamic exchange of membranes, which is necessary for dendrites to reliably maintain Ca^2+^ homeostasis for extended durations [13,14]. The stacked-sheet architecture also permits an exceptionally high density of ER membrane in cell bodies, which provides an expansive surface for ribosome attachment [4]. Attachment of ribosomes to ER sheets is necessary for protein translation, and morphologically distinguishes rough ER (RER) from smooth ER (SER). Somatodendritic compartments contain both SER and RER, which exhibit dynamic transitions between each other [15].

In contrast to the sheet-like structures in cell bodies and dendrites, axonal ER is comprised of thin (~20–30 nm in diameter), elongated, and smooth tubules [3,16]. RER constitutes a very small fraction of axonal ER [17]. The relative paucity of RER in axons suggests a diminished contribution of ER-associated ribosomes in axonal protein translation. ER tubules are sculpted from sheets by the complex interplay between many proteins including atlastin, the reticulon complex, and members of the receptor expression enhancing proteins (REEPs) [7,18,19,20,21]. Once tubulated, growth of nascent ER along microtubules allows the organelle to populate the entire length of the axon [22]. The axonal ER and microtubules exhibit multiple levels of crosstalk, and function together to mediate axonal elongation and neuronal polarity [20,22,23]. In addition to microtubules, neurofilaments and atypical myosins (e.g., myosin Va) also regulate the shape and distribution of axonal ER, which in return guides axonal growth [24,25].

## 3. ER-Resident Ca^2+^ Channels Are Required for the Regulation of Axodendritic Growth and Morphology

**Figure 1:**
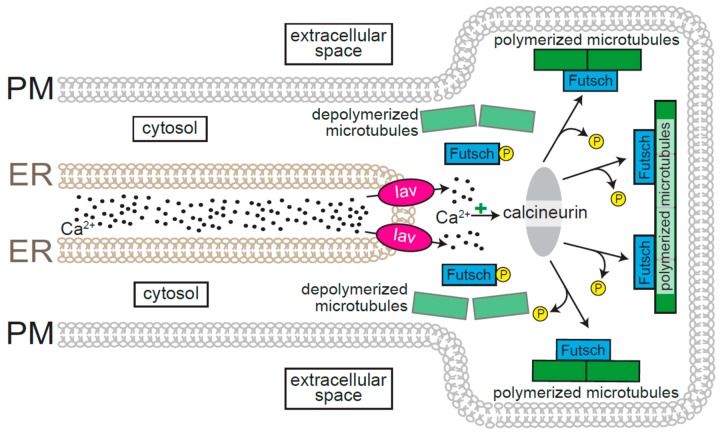
Schematic depicting the role of the ER Ca^2+^ channel, Inactive (Iav), in the formation of *Drosophila* presynaptic boutons. Within the *Drosophila* neuromuscular junction, presynaptic boutons morphology and number are regulated by stabilized microtubules. Phosphorylated Futsch cannot bind to microtubules, leading to microtubule destabilization. The Ca^2+^-dependent phosphatase, calcineurin, is activated by Ca^2+^ released by Iav and subsequently dephosphorylates Futsch, which leads to microtubule stabilization.

As mentioned at the end of the previous section, the relationship between the structures of neurons and the underlying ER is inherently bidirectional. While neuronal architecture imposes specific constraints on ER morphology, ER Ca^2+^ release and the channels involved therein influence the structure of neurons. Analyses of invertebrate synapses have revealed several aspects of the interplay between Ca^2+^ and neuronal structure and function. Since the sites of neurotransmitter release are located within clearly identifiable presynaptic boutons at *Drosophila* neuromuscular junctions (NMJ) [26,27,28], determinants of bouton morphology and number at these synapses have been matters of intense study. The stereotypical arborization of axon termini at *Drosophila* NMJs is strongly influenced by the stability of presynaptic microtubules, which depends on the microtubule binding protein 1b (MAP1b) homolog, Futsch (Figure 1) [29,30,31]. Futsch–microtubule association promotes microtubule stability, whereas diminished interaction with Futsch results in microtubule depolymerization and stereotypical alterations in NMJ morphology—larger but fewer boutons [23,29,30,31]. As is the case for many microtubule binding proteins, Futsch–microtubule association is inversely correlated with Futsch phosphorylation [32]. Ca^2+^ release via the ER-resident transient receptor potential vanilloid (TRPV) channel, Inactive (Iav) [33,34], leads to activation of the Ca^2+^-dependent phosphatase, calcineurin (Figure 1) [23]. Once activated, calcineurin mediates Futsch dephosphorylation, increased microtubule stability, and normal bouton morphology [23]. Decreased abundance of Iav and *Drosophila* homologs of the ER channels, inositol triphosphate receptors (IP_3_Rs), and ryanodine receptors (RyRs), results in diminished calcineurin activity, loss of presynaptic microtubules, and attendant alterations in bouton number and morphology [23]. The observation that activities of Iav, IP_3_R, and RyR are required for regulation of presynaptic morphology points to functional interactions between these channels in the regulation of ER Ca^2+^ release and synapse development. 

ER Ca^2+^ and microtubules are also involved in axon guidance [23]. Ca^2+^ release via RyRs, and the subsequent activation of store-operated channels mediate the effects of netrin-1 on growth cone guidance [35,36,37,38]. IP_3_Rs also influence netrin-1-dependent turning by regulating the rate of microtubule invasion into growth cones [37]. Irrespective of the mechanism of store depletion, the luminal [Ca^2+^] sensor, stromal interacting molecule 1 (STIM1), couples store release with microtubule polymerization via interactions with the microtubule plus-end binding protein, EB1 [36,39,40,41,42]. Furthermore, membranes necessary for neurite extension are derived from ER Ca^2+^-dependent asymmetric secretion of vesicles towards the growing end of axons [36,39,40,41,42]. The movement and fusion of these vesicles with the axonal membrane depend on myosin Va, and are facilitated by activation of IP_3_Rs or RyRs [24,25]. Myosin Va is also necessary for maintaining the appropriate distribution of ER in axonal periphery, without which proper axonal growth would be impeded [24,25].

STIM and ER [Ca^2+^] also regulate the growth and morphology of dendritic spines. Neuronal activity-dependent modification of the spine structure during long-term potentiation involves ER Ca^2+^ store depletion, STIM1-mediated induction of store-operated Ca^2+^ entry, and gene expression driven by the transcription factor NFATc3 [43,44]. In hippocampal neurons, directional movement of microtubules into spines, an intermediary in the regulation of spine morphology by ER Ca^2+^ release, depends on interactions between STIM2 and the microtubule plus-end binding protein EB3 [45]. As is the case in axons, release of ER Ca^2+^ within synaptic spines is predicated on the presence of ER. Myosin Va-dependent movement of ER along actin fibers drags the organelle into growing spines [46,47], thus facilitating spine development via pathways involving ER Ca^2+^ release. Together, these findings suggest that actomyosins and microtubules influence temporally distinguishable events that sculpt neuronal morphology. Myosin Va appears to set the stage by ensuring the availability of ER in the relevant compartments such that subsequent release of ER Ca^2+^ facilitates local remodeling in a microtubule-dependent manner. 

## 4. ER Ca^2+^ and the Regulation of Neurotransmission

**Figure 2:**
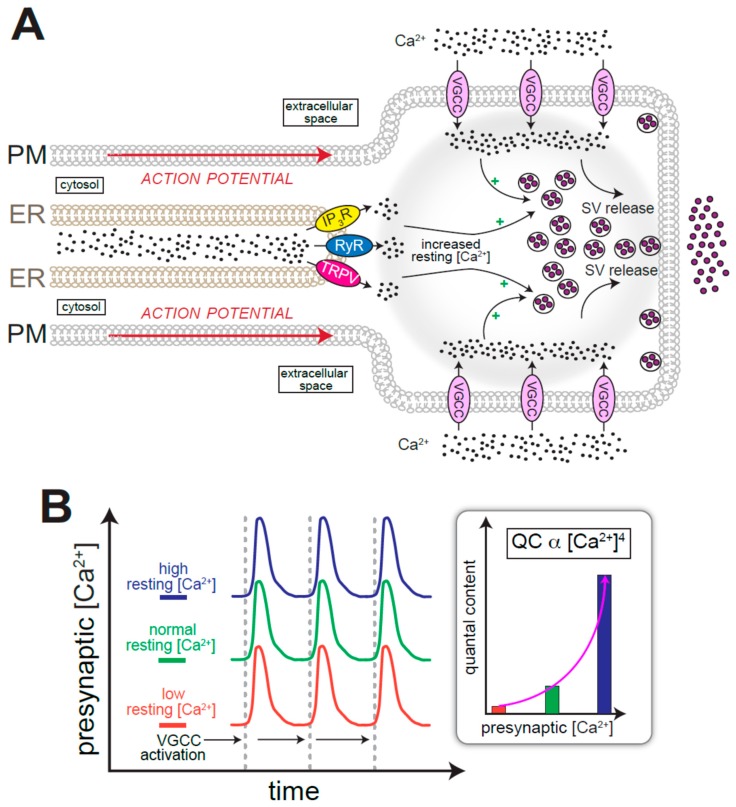
Influence of ER Ca^2+^ release on synaptic vesicle (SV) release and neurotransmission. (**A**) Action potentials are propagated through axons. Depolarization of presynaptic membrane activates voltage-gated Ca^2+^ channels (VGCCs), which causes Ca^2+^ to enter the synapse. ER Ca^2+^ release contributes to cytosolic [Ca^2+^], which influences release of neurotransmitter-laden SVs. (**B**) ER Ca^2+^ release, whether increased or decreased, affects the presynaptic cytosolic [Ca^2+^]. The presynaptic cytosolic [Ca^2+^] scales linearly upon activation of VGCCs. The magnitude of SV release obeys a 4th power relationship with presynaptic cytosolic [Ca^2+^], allowing small elevations in [Ca^2+^] to cause large increases in neurotransmitter release (inset).

Axons contain the domains responsible for the release of neurotransmitter-laden synaptic vesicles (SVs). The fundamentals of SV release, which are reasonably well-understood, can be succinctly summarized as a sequence of events starting with the arrival of action potentials to presynaptic termini, opening of voltage-gated Ca^2+^ channels (VGCCs), Ca^2+^ influx, and Ca^2+^/SNARE-dependent SV exocytosis (Figure 2A) [48,49]. According to this model, instructive signals that elevate presynaptic [Ca^2+^] to a range necessary for SV release are plasma membrane delimited, i.e., channels responsible for both action potential propagation and Ca^2+^ entry are located on the cell surface. Nevertheless, many studies have demonstrated the involvement of ER Ca^2+^ channels in sculpting the temporal dynamics and extent of SV release. The probability of SV release, defined as the likelihood that a primed SV will be released upon arrival of the action potential, is influenced by presynaptic resting [Ca^2+^] (Figure 2B) [23,49,50,51,52]. Given the 4^th^ power relationship between [Ca^2+^] and quantal content (referred to as the nonlinear relationship between Ca^2+^ and vesicle release rates [53]), small changes in resting [Ca^2+^] exert considerable influence on neurotransmitter release (Figure 2B, inset) [23,49,50,51]. At the calyx of Held, for instance, even a 50 nM increase in resting [Ca^2+^] is sufficient to elicit an appreciable increase in SV release probability [51]. Many different processes, including Ca^2+^ entry and extrusion mechanisms, cooperate to determine presynaptic resting [Ca^2+^] [50]. Roles for ER Ca^2+^ and ER-resident channels in crafting resting [Ca^2+^] and SV release probability have been widely acknowledged (Figure 2A) [23,54,55,56,57,58,59,60,61,62]. At the hippocampal Schaffer-collateral pathway, deletion of presenilin in presynaptic CA3 neurons perturbs short-term plasticity via hyperactivation of RyRs [54,59]. Elevations in RyR-mediated ER Ca^2+^ release promote both spontaneous SV release and the probability of evoked events [55,63,64]. Furthermore, either the induction of passive leak of ER Ca^2+^ following inhibition of the Ca^2+^ ATPase, SERCA, or activation of IP_3_R mediated ER Ca^2+^ release are sufficient for presynaptic facilitation [54,65,66]. These data point to a role for ER Ca^2+^ release per se rather than an obligatory role for any specific channel type. Needless to say, the influence of ER Ca^2+^ release on overall network dynamics depends on the identity of the neurons experiencing the Ca^2+^ release events, as exemplified by the findings that increased RyR activity or inhibition of SERCA elevates the probability of GABA release in Purkinje neurons, which serves to dampen network activity in the cerebellum [56,57]. 

Although several studies have shown that Ca^2+^-induced Ca^2+^ release (CICR, mediated by either RyR or IP_3_R) is necessary for ER stores to adequately influence presynaptic plasticity and SV release, the notion of CICR influencing SV release has incompletely understood nuances [54,55,61,62]. For instance, other studies argue in favor of a model that residual Ca^2+^ from VGCC opening, and not CICR, is the major regulator of resting [Ca^2+^] and presynaptic plasticity in cerebellar and hippocampal slices [67]. A potential explanation for these conflicting viewpoints could be gleaned from studies focused on the role of ER Ca^2+^ release in presynaptic termini of *Drosophila* NMJs. Loss-of-function mutations in the gene encoding the TRPV channel, Iav, result in lower presynaptic resting [Ca^2+^] and decreased SV release probability [23]. Interestingly, these phenotypes were only evident when the extracellular [Ca^2+^] was lowered to <0.5 mM from the usual 2 mM [23]. These phenotypes, which have been recapitulated in other mutants with diminished ER Ca^2+^ release at the *Drosophila* larval NMJ [20,68], show that the contribution of ER channels on resting [Ca^2+^] can be overridden by Ca^2+^ influx. We envision that only during periods of diminished presynaptic Ca^2+^ entry would the contribution of ER Ca^2+^ stores to SV release become apparent. For example, during high frequency stimulation when massive Ca^2+^ influx into postsynaptic neurons results in dramatic reduction of [Ca^2+^] in the synaptic cleft [69,70,71,72], ER Ca^2+^ release could have a significantly larger impact on determining resting [Ca^2+^], and thus SV exocytosis. 

## 5. ER Ca^2+^ Release Regulates Neuronal Gene Expression during Development, Synaptic Plasticity, and Cell Death 

While axons and dendrites participate in intercellular communication, cell bodies contain the nuclei, and thus serve as the seat of neuronal gene expression. Ca^2+^ is a key intermediate in the functional relationship between neuronal activity and gene expression. Alterations in cytosolic and nuclear [Ca^2+^] exert differential effects on neuronal gene expression, the dynamics of which are synchronized with the spatiotemporal patterns of the Ca^2+^ transients. Depletion of ER Ca^2+^ stores by application of the SERCA inhibitor, thapsigargin, in cortical, hippocampal, and other neurons leads to the dramatic and rapid increase in the transcription of the immediate early genes encoding c-Fos and Homer-1a [73,74]. Stimulation of ER Ca^2+^ release in hypothalamic neurons following application of serotonin also results in c-Fos induction via a process requiring ER Ca^2+^ release through IP_3_Rs [75,76]. The relationship between c-Fos induction and ER Ca^2+^ release reinforces the role for the latter in synaptic plasticity. Furthermore, CREB-dependent gene expression, which is also critically important for synaptic plasticity, is influenced by different sources of neuronal Ca^2+^ elevation [77,78,79]. Indeed, c-Fos expression is itself dependent on CREB, potentially via IP_3_R-dependent nuclear translocation of a CREB coactivator called TORC1 (not to be confused with the kinase complex containing mTOR) [76,80]. IP_3_ production and ER Ca^2+^ release via IP_3_Rs during synaptic plasticity is often triggered by stimulation of metabotropic glutamate receptors [81]. It is notable that in the context of synaptic plasticity, elevation of nuclear [Ca^2+^] arising from the passive diffusion from the cytosol through the nuclear pore or due to activation of IP_3_Rs embedded in the nuclear envelope promotes CREB-dependent transcription and the changes in synaptic strength and dendritic arborization that characterize plasticity [82,83,84,85,86,87,88,89]. 

**Figure 3:**
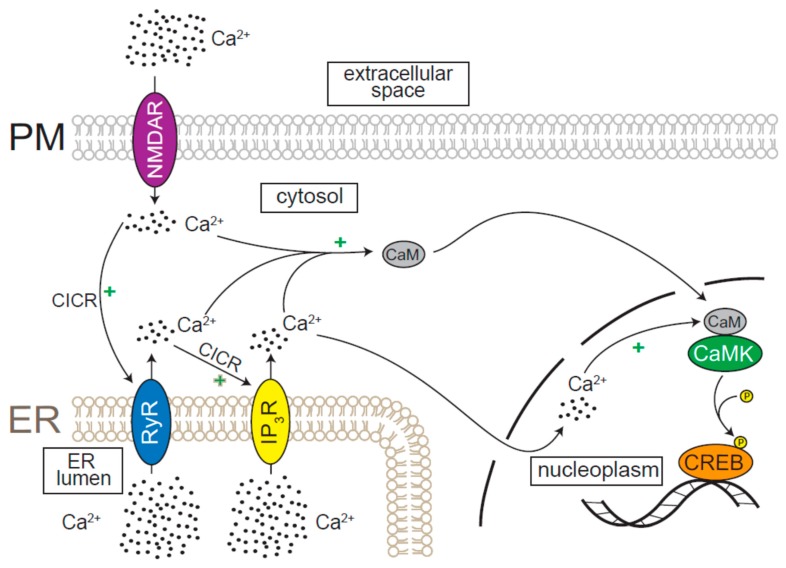
Ca^2+^-mediated activation of neuronal gene expression. Dendritic stimulation activates channels on the plasma membrane (PM), such as the N-methyl-D-aspartate receptor (NMDAR), which allow Ca^2+^ influx. RyR responds to Ca^2+^ entry and the signal is amplified through Ca^2+^-induced Ca^2+^ release (CICR). Subsequent activation of IP_3_R causes further Ca^2+^ release and activation of calmodulin (CaM). CaM may be activated either directly in the nucleus or in the cytosol, in which case it translocates to the nucleus, and activates Ca^2+^/calmodulin-dependent protein kinases (CaMKs). CaMKs phosphorylate cAMP response element binding (CREB) protein, which binds to DNA and drives changes in gene expression.

The canonical mode of CREB activation in response to cytosolic or nuclear Ca^2+^ elevation involves CREB phosphorylation by Ca^2+^/calmodulin-responsive enzymes, CaMKII and CaMKIV (Figure 3) [79,90,91]. The elevation of [Ca^2+^] that precedes CaMK- and CREB-dependent synaptic plasticity is triggered by synaptic transmission occurring at dendritic spines, which begs the question: how are signals transmitted from dendrites to the nucleus? One explanation for this long-range spatial communication is that dendritic Ca^2+^ elevations result in CICR (RyR-dependent) and the propagation of ER Ca^2+^ waves (likely IP_3_R-dependent), ultimately resulting the elevation of nuclear Ca^2+^ and CREB-dependent gene expression (Figure 3) [83,92]. 

In T lymphocytes, NFAT-dependent gene expression is predicated upon the movement of the transcription factor from the cytosol to nucleus [93,94,95,96]. As phosphorylation inhibits nucleocytoplasmic transport of NFAT, calcineurin activated by ER Ca^2+^ release and store-operated Ca^2+^ entry is needed for NFAT dephosphorylation and attendant gene expression [93,94,95,96]. Similarly, cytosolic Ca^2+^ elevations in neurons lead to NFAT-dependent gene transcription in a calcineurin-dependent manner [97,98]. In addition to Ca^2+^ entry accompanying neuronal activity, IP_3_R-mediated ER Ca^2+^ release downstream of BDNF application or alterations in mitochondrial Ca^2+^ uptake drive nuclear translocation of NFAT in neurons [97,98,99]. Whereas calcineurin/NFAT participate in neurodevelopment and synaptic plasticity in some contexts [43,100,101], activation of this transcriptional axis by elevated Ca^2+^ release in mature neurons usually promotes cell death [102,103]. Furthermore, alterations in ER Ca^2+^ homeostasis is also thought to be a determinant of gene expression changes observed in aged neurons, and could involve calcineurin and NFAT [104]. Interestingly, expression of the genes encoding IP_3_R1 and IP_3_R2 are under the control of calcineurin and NFAT [105,106,107,108], which prompts us to speculate that elevated channel activity could indirectly promote its abundance, and thus compromise neuronal viability during aging and disease.

## 6. Relationship of ER Ca^2+^ Release with Neuronal Bioenergetics and Excitability

**Figure 4:**
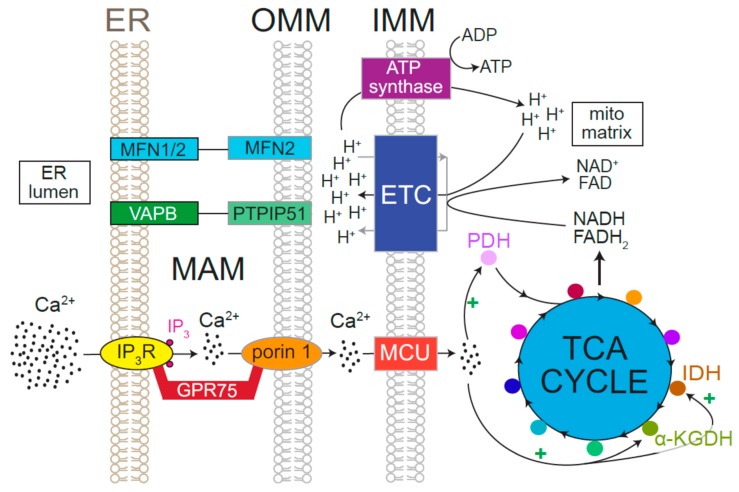
ER Ca^2+^ release stimulates oxidative phosphorylation. Mitochondria-associated membranes (MAMs) are points of contact between the ER and mitochondria that have various functions, including Ca^2+^ signaling between the two organelles. MAMs are stabilized by protein pairs that bind to each other, serving as molecular tethers. The VAPB-PTPIP51 and MFN1/2-MFN2 binding pairs are two such examples. IP_3_R exists in MAMs and is linked via GPR75 to porin 1, which is found in the outer mitochondrial membrane (OMM). In this arrangement, IP_3_-mediated Ca^2+^ release enables efficient transfer of Ca^2+^ into the intermembrane space, where the low-affinity mitochondrial Ca^2+^ uniporter (MCU) can be overcome by concentrated Ca^2+^ microdomains. Ca^2+^ that enters the mitochondrial matrix stimulates the tricarboxylic acid (TCA) cycle by activating pyruvate-dehydrogenase (PDH), isocitrate-dehydrogenase (IDH), and alpha-ketoglutarate dehydrogenase (KGDH). The TCA cycles produces the reducing equivalents (NADH and FADH_2_) that power the electron transport chain (ETC), which produces a protein gradient in the intermembrane space. Protons (H^+^) flow down their concentration gradient through ATP synthase leading to biogenesis of ATP.

**Figure 5:**
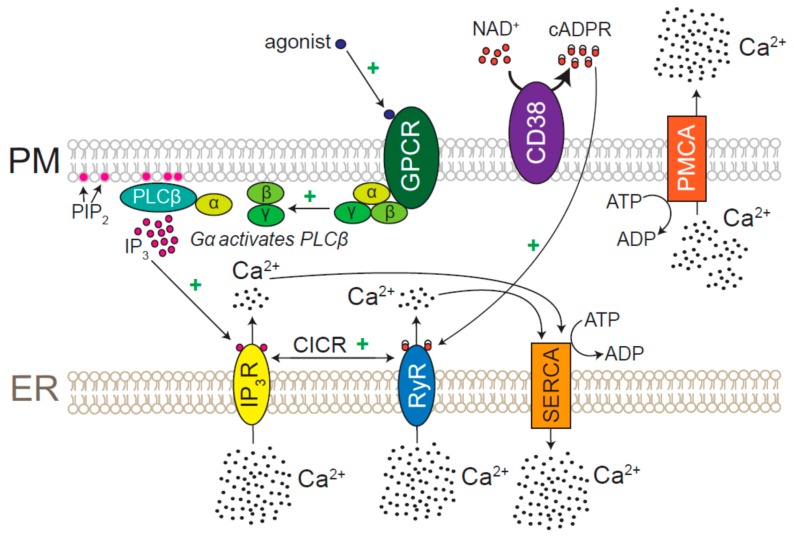
Mechanisms of ER channel activation. Activation of IP_3_R begins at the plasma membrane (PM), where agonist activation of G-protein coupled receptors (GPCRs) leads to dissociation of heterotrimeric Gq protein. Gq activates PLC, which cleaves phosphatidylinositol 4,5-bisphosphate (PIP_2_) into inositol trisphosphate (IP_3_) and diacylglycerol (DAG). IP_3_ serves as a second messenger and activates IP_3_R, causing ER Ca^2+^ release. Alternatively, RyR is activated by cyclic ADP ribose (cADPR), which is synthesized in the extracellular space from nicotinamide adenine dinucleotide (NAD^+^) by the ectoenzyme, CD38. Both IP_3_R and RyR are themselves activated by Ca^2+^ and can stimulate each via Ca^2+^-induced Ca^2+^ release (CICR). The resulting increase in cytosolic Ca^2+^ is removed by either plasma membrane Ca^2+^ ATPase (PMCA) or sarco/endoplasmic reticulum Ca^2+^-ATPase (SERCA), which pump Ca^2+^ into the extracellular space or ER, respectively.

The healthy brain is a veritable powerhouse, a fact that is best illustrated by the involvement of this organ in the production and consumption of ~20% of an individual’s energy despite contributing only ~2% to the total body mass [109]. Within the brain, most of the bioenergetic activity occurs in neurons, which utilize ATP generated from mitochondrial oxidative phosphorylation (OXPHOS) for a variety of key functions. The reducing equivalents that power OXPHOS are produced by the tricarboxylic acid (TCA) cycle in the mitochondrial matrix [110]. While it is widely acknowledged that the TCA cycle requires metabolites such as pyruvate and α-ketoglutarate [111,112,113], a less appreciated fact is that >20 μM [Ca^2+^] is needed within the matrix for activation of the TCA dehydrogenases—pyruvate dehydrogenase, α-ketoglutarate dehydrogenase, and isocitrate dehydrogenase (Figure 4) [113]. This requirement necessitates an acute elevation of matrix [Ca^2+^] from the resting levels of ~500 nM, which is made challenging by the relatively low Ca^2+^ affinity (20–30 μM) of the mitochondrial Ca^2+^ uniporter (MCU) [114]. Within the presynaptic compartment, routine machinations of neurotransmission elevate cytosolic [Ca^2+^] to levels high enough for mitochondrial uptake and the attendant activation of OXPHOS [114]. At these sites, locally produced ATP is immediately made available to plasma membrane Ca^2+^ ATPases (PMCA), which use this source of energy to actively extrude intrasynaptic Ca^2+^ (Figure 5) [115,116]. The situation is substantially more complex in cell bodies where the threat of Ca^2+^ cytotoxicity precludes sustained [Ca^2+^] elevation in the bulk cytosol. To overcome these restrictive conditions, a fraction of ER is physically associated with mitochondria allowing the two organelles to form specialized structures called mitochondrial associated membranes (MAMs) [117,118]. Amongst their myriad functions, MAMs are characterized by the presence of IP_3_Rs, and thus serve as conduits for interorganellar Ca^2+^ transfer [117,119,120,121,122,123,124]. ER Ca^2+^ release by IP_3_Rs in MAMs enables sufficient elevation of perimitochondrial [Ca^2+^] and MCU-mediated Ca^2+^ uptake [119]. IP_3_Rs are maintained in MAMs by physical tethering to the outer mitochondrial membrane-resident protein, porin1, with GRP75 serving as an intermediary link (Figure 4) [119]. These interactions allow IP_3_Rs to sculpt both cytosolic and mitochondrial [Ca^2+^], with the latter playing a major role in the regulation of cellular bioenergetics. It is important to note that despite the definitive involvement of MAMs in interorganellar Ca^2+^ transfer, cytosolic [Ca^2+^] elevations that exceed the MCU threshold would increase mitochondrial [Ca^2+^] in a manner that is agnostic to the source of Ca^2+^. For instance, cytosolic [Ca^2+^] elevations subsequent to RyR activation also result in mitochondrial Ca^2+^ uptake, albeit in a MAM-independent manner [125,126]. Concordantly, pharmacological inhibition of RyRs are beneficial in neurological diseases associated with Ca^2+^-dependent mitochondrial dysfunction [127]. 

The extent of energy production in neurons is intimately related to neuronal excitability. Almost all the processes involved in the regulation of neuronal membrane potential, excitability, and propagation of action potentials exhibit steep dependences on [ATP]. Thus, the interplay between IP_3_R activation and mitochondrial ATP production ensures a critical requirement for IP_3_Rs in the dynamic regulation of neuronal membrane potential and excitability. This notion is exemplified by the Na^+^/K^+^ ATPase, which is needed for the maintenance of the electrical polarity across the neuronal membrane, and for this purpose the pump consumes ~75%–90% of the ATP produced in the cell [128]. Computational studies modeling the relationships between OXPHOS and neuronal excitability point to the involvement of the Na^+^/K^+^ ATPase in the susceptibility of neurons to ATP deprivation [129]. In this schema, even a 15% deficit in [ATP] is expected to result in fulminant loss of neuronal membrane potential due to the progressive decline in Na^+^/K^+^ ATPase activity [129]. Thus, it stands to reason that alterations in ER Ca^2+^ release are reflected in the extent of ATP production and membrane potential. In addition, dynamic changes in neuronal excitability are also subject to regulation by ER Ca^2+^ channels. For example, in cortical neurons previously treated with caged IP_3_, action potentials were consistently stronger [130]. Along these lines, the Ca^2+^ store-responsive protein, STIM1, has been shown to play a role in ATP production and the resulting regulation of Ca^2+^ extrusion and the intrinsic firing properties of Purkinje neurons [131]. On the other hand, Ca^2+^ release via either RyRs or IP_3_Rs can activate Ca^2+^-responsive K^+^ channels (e.g., BK channels), which hyperpolarize neurons and repress excitability [132]. Together, these findings implicate several interlocking functions of ER Ca^2+^ channels in the regulation of neuronal excitability.

## 7. Mechanisms Involved in the Activation of ER Ca^2+^ Release in Neurons

### 7.1. Role for CICR and Ca^2+^ Pumps in ER Ca^2+^ Homeostasis

Since IP_3_R-dependent cytosolic Ca^2+^ elevation is usually sufficient for stimulating RyRs, IP_3_Rs and RyRs are thought to cooperate in the initiation and propagation of CICR [133,134,135,136]. Both types of channels exhibit biphasic dependence on cytosolic [Ca^2+^] owing to the presence of high Ca^2+^-affinity activating sites and low Ca^2+^-affinity inhibitory sites on the proteins [137,138,139,140]. When cytosolic [Ca^2+^] is below a critical threshold, Ca^2+^ ions activate the channels leading to store release. Upon exceeding the threshold, Ca^2+^ ions start to inhibit the channels, which lead to attenuation of Ca^2+^ release and restoration of cytosolic [Ca^2+^] to resting levels. In addition to IP_3_Rs and RyRs, other purported triggers for CICR include Ca^2+^ influx via TRP channels and VGCCs, and alterations in mitochondrial Ca^2+^ uptake [141,142,143,144]. Given the inherently self-sustaining nature of CICR, functional crosstalk between RyRs and other modulators of cellular Ca^2+^ homeostasis permits the amplification of even small or transient elevations in cytosolic [Ca^2+^]. 

ER Ca^2+^ release is also coupled to the mechanisms of Ca^2+^ removal. Whereas extrusion of Ca^2+^ by PMCA serves to lower cytosolic [Ca^2+^] and limit CICR, Ca^2+^ uptake into the ER promotes subsequent release [60,144]. Given the 10^4^-fold [Ca^2+^] gradient between the ER lumen and the cytosol, the overall Ca^2+^ content of the ER serves as a powerful driving force for Ca^2+^ release [60]. Neuronal activity results in SERCA-dependent sequestration of Ca^2+^ into the ER stores, which when released, promotes store-operated Ca^2+^ entry and modulates SV release probability [60]. These findings demonstrate the functional relationships between plasma membrane-resident channels, Ca^2+^ pumps, and ER channels in the regulation of neuronal Ca^2+^ homeostasis. 

### 7.2. Physiological Agonists of IP_3_Rs and RyRs

IP_3_R1 and RyR1/RyR3 are the respective channel isoforms that are most enriched in the nervous system [145,146]. Despite exhibiting qualitatively similar relationships with Ca^2+^, the two families of channels differ markedly in biophysical parameters such as ionic conductance, adaptation and inactivation mechanisms, and kinetics [136]. IP_3_Rs are activated by the soluble second messenger, IP_3_, which lowers the apparent Ca^2+^ responsiveness of the channels towards resting cytosolic [Ca^2+^] [147,148]. Phospholipase C (PLC) enzymes, predominantly comprised of PLCβ, PLCγ, and PLCδ subtypes, hydrolyze the phosphoinositide, PI(4,5)P_2_ (henceforth referred to as PIP_2_), to generate diacylglycerol (DAG) and IP_3_ (Figure 5) [147,149,150]. Although all three subtypes of PLC hydrolyze PIP_2_, they exhibit distinct activation and inactivation kinetics such that the shapes and dynamics of the attendant ER Ca^2+^ release transients are a function of the subtype being stimulated. Whereas PLCβ isoforms are activated by Gq-coupled receptors, PLCγ isoforms are coupled to receptor tyrosine kinase signaling pathways, and PLCδ isoforms are regulated by phospholipids and Ca^2+^ [149,150,151,152,153]. The enzyme subtypes also have distinct functions in the nervous system. For instance, PLCβ activation is involved in regulation of neuronal tone and excitability, whereas PLCγ activation has a bigger influence on neurodevelopment and synaptic plasticity [38,150,154,155,156,157,158,159]. It is important to note, however, that only a subset of PLC functions can be attributed to subsequent IP_3_R activation since PLC-mediated PIP_2_ depletion can influence neuronal Ca^2+^ homeostasis via a number of different pathways [34]. IP_3_ kinases, IP_3_K1 and IP_3_K2, convert IP_3_ to the higher inositol phosphate, IP_4_, which is unable to bind IP_3_Rs [160]. Thus, the combined activities of IP_3_K1 and IP_3_K2 serve to restrict channel activity.

RyR activity is modulated by ryanodine and Ca^2+^, and promoted by the second messenger, cyclic ADP ribose (cADPR), synthesized from NAD^+^ by ADP-ribosyl cyclases such as CD38 and CD157 (Figure 5) [161,162,163,164,165]. Depending upon the context and cell type, RyR activation by cADPR could also involve accessory proteins such as calmodulin [166,167,168]. Not surprisingly, many functions of RyRs in neurons are subject to regulation by ADP-ribosyl cyclases and cADPR [148]. For instance, cADPR-mediated release of ER Ca^2+^ affects the excitability of dorsal root ganglion (DRG) neurons via Ca^2+^-activated currents [169,170]. Activation of RyRs by cADPR is also involved in the regulation of neuronal firing frequency, SV release, and synaptic plasticity in both vertebrate and invertebrate neurons [171,172,173,174]. 

### 7.3. Mechanisms Involved in the Activation of ER-Resident TRP Channels

TRPVs, TRPM1, TRPM8, and TRPP2 subtypes of the TRP superfamily of cation channels have also been shown to localize and/or function in ER Ca^2+^ release [175,176,177,178,179]. TRPP2 channels have been suggested to function as Ca^2+^-activated release channels, although this finding needs further verification [177,178]. While TRPM8 activation in the ER has been suggested to require phosphoinositides [176], the activation mechanisms for ER-resident TRPM1 remain unknown. As mentioned previously, the *Drosophila* TRPV channel, Iav, is localized to axonal and presynaptic ER in motor neurons [23,180]. Iav-mediated ER Ca^2+^ release is required for the maintenance of presynaptic resting [Ca^2+^] and SV release probability [23]. The localization and function of a *Drosophila* TRPV channel in the ER is reminiscent of a fraction of mammalian TRPV1 being localized to the ER, and in close proximity to IP_3_Rs [181,182,183,184,185,186,187,188,189]. Concordantly, either the overexpression of TRPV1 or application of the channel agonist, capsaicin, leads to ER Ca^2+^ release [23]. Involvement of TRPV1 in ER Ca^2+^ release also explains why ectopic expression of TRPV1, but not TRPV4, in *Drosophila* motor neurons rescued the defects associated with ER Ca^2+^ release in hypomorphic alleles of the gene encoding Iav [183,184,185,186,187,188,189]. The exact mechanisms involved in the stimulation of TRPV1 or Iav in the ER remain unknown. Given the activation of TRPV1 by heat and endocannabinoids, it is possible that these modalities also activate the channel in the ER. Iav activity is regulated by mechanical stretch [181,190], although the involvement of stretch in Iav-mediated ER Ca^2+^ release has not been examined. We speculate that the role of Iav and TRPV1 in the regulation of resting [Ca^2+^] could reflect a potential mode of activation. Both Iav and TRPV1 contain Ca^2+^/calmodulin-binding motifs in their cytosolic domains. In case of TRPV1, occupancy of these sites by Ca^2+^/calmodulin leads to channel desensitization [33]. Notably, deletion of the calmodulin-binding site on TRPV1 potentiates capsaicin-induced ER Ca^2+^ release, which points to a role for Ca^2+^/calmodulin in channel inactivation at the ER [191,192]. It is possible that a similar mechanism suppresses Iav at resting or higher cytosolic [Ca^2+^]. If so, a drop in cytosolic [Ca^2+^] would disinhibit Iav, leading to ER Ca^2+^ release. We predict that this axis serves as a feedback mechanism for the maintenance of resting [Ca^2+^] within a narrow physiologically important level at presynaptic termini. 

## 8. Pathologies Associated with Neuronal ER Ca^2+^ Dyshomeostasis

Perturbed Ca^2+^ homeostasis occurs in many neurological diseases. Being the largest intracellular reservoir of Ca^2+^, ER Ca^2+^ dyshomeostasis is of particular relevance to these diseases. In this section, we present an overview of the relationships between ER Ca^2+^ and pathology in a sampling of neurological diseases. We hope to highlight the tenet that qualitatively distinct alterations in ER Ca^2+^ signaling (either too much or too little release or uptake) can result in pathology. In fact, ostensibly similar changes in ER Ca^2+^ homeostasis can induce diverse spatiotemporal outcomes, indicating that changes in ER Ca^2+^ dynamics accompany neuronal processes perturbed in disease. 

### 8.1. Autism Spectrum Disorder (ASD)

ASD is a constellation of neurodevelopmental disorders characterized by limited interpersonal communication and social skills. Although the molecular etiology of ASD is highly complex, evidence points to the involvement of ER Ca^2+^ alterations in its pathophysiology [193]. In cells derived from patients suffering from fragile X syndrome and tuberous sclerosis, which are monogenic syndromes that present with ASD, IP_3_-mediated Ca^2+^ signaling is compromised [194]. This paradigm has also been extended to sporadic forms of ASD that are characterized by diminished ER Ca^2+^ release in response to stimulation of PLCβ-coupled receptors [195]. The purported involvement of IP_3_Rs in ASD likely stems from the role of these channels in the regulation of neuronal excitability and the excitation–inhibition balance of neuronal circuits, both of which are necessary for the regulation of social behaviors [193].

Mutations in *Ryr1* accompany enhanced dendritic arborization and impaired social behavior in mice [196]. Behavioral analyses of *Ryr3*-deficient mice also suggested abnormal social interactions [197]. The relationship between RyRs and social behavior is not restricted to mice since a gene duplication has pointed to *RYR2* as a target gene in a Lebanese cohort of ASD patients [198]. Additional insights into ER Ca^2+^ dyshomeostasis in ASD may be gleaned from studies of fragile X, in which loss of fragile X mental retardation protein (FMRP) results in the broadening of action potential waveforms, and elevated neurotransmitter release [199]. The involvement of Ca^2+^-activated BK-type K^+^ channels in this process raises the possibility that the demonstrated relationship between BK channels and ER Ca^2+^ release could contribute to autistic behaviors [132,199].

### 8.2. Lysosomal Storage Diseases (LSDs)

LSDs are inborn errors of metabolism that are often associated with severe neurodevelopmental defects [200]. Although primarily associated with endolysosomal dysfunction, sustained changes in ER Ca^2+^ dynamics have been reported in several LSDs [201,202,203,204,205,206,207]. The relationship between lysosomes and ER in the context of Ca^2+^ homeostasis agrees with the findings that endolysosomal Ca^2+^ can be traced to the ER. Ca^2+^ released by activated IP_3_Rs is loaded into lysosomes, which in turn, mediates various aspects of endolysosomal function [208,209]. Although the mechanisms of endolysosomal Ca^2+^ uptake remain incompletely understood, involvement of vesicular K^+^ channels or Ca^2+^/H^+^ exchangers have been suggested [210,211,212]. Reminiscent of MAMs that from between ER and mitochondria, organellar contact sites between the ER and endolysosomes form in a VPS13C-dependent manner, and permit localized [Ca^2+^] elevations needed for interorganellar ion transfer [208,213,214]. 

The aforementioned relationships between ER and lysosomal Ca^2+^ also explain some of the pathological features of LSDs. For instance, in primary neuronal cultures generated from murine models of Gaucher’s disease and patient-derived tissues, accumulation of glucosylceramide potentiated RyRs resulting in excessive Ca^2+^ release [201,202]. Concordantly, increased expression of *SERCA2b* or inhibition of RyR, two strategies that increase ER luminal Ca^2+^, improved proteostasis and the stability of glucocerebrosidase, the lysosomal enzyme mutated in Gaucher’s disease [203]. These findings suggest that elevated ER Ca^2+^ release is a pathological event in Gaucher’s disease, likely triggering the unfolded protein response due to sustained ER Ca^2+^ store depletion [204]. RyR antagonists were also found to be beneficial in restoring lipid homeostasis in cells isolated from patients suffering from Niemann-Pick type C [205]. Similarly, in murine models of GM1-gangliosidosis, the accumulation of GM1-ganglioside within MAMs results in the activation of IP_3_R1 and attendant ER Ca^2+^ depletion [206], whereas in Sandhoff’s disease (caused by deletion of *HexB*), GM2-gangliosides inhibit SERCA, leading to the passive depletion of ER Ca^2+^ stores [207]. Together, these studies prompt the speculation that suppression of ER Ca^2+^ release mechanisms could restore proteostasis, and thereby prove beneficial in LSDs.

### 8.3. Neuropsychiatric Diseases

In accordance with the involvement of ER Ca^2+^ dynamics in neuronal development, synaptic plasticity, and excitability, Ca^2+^ dyshomeostasis has been described in neuropsychiatric diseases [215]. Darier’s disease, which is caused by mutations in the SERCA2 encoding *ATP2A2* gene, demonstrates a causal link between ER Ca^2+^ and neuropsychiatric disorders [215]. Amongst the spectrum of pathophysiological features in Darier’s disease is the prevalence of schizophrenia, bipolar disorder, and major depression [215,216,217,218,219,220]. Interestingly, Darier’s patients exhibit distinct neuropsychiatric outcomes depending on the locations of the mutations in SERCA2 [215]. Consistent with the appearance of psychiatric symptoms in Darier’s patients, it is notable that ER stress, an established outcome of sustained Ca^2+^ store depletion, is associated with the neuroplastic changes observed in major depression [221]. This notion informs the idea that attenuation of ER Ca^2+^ release would counteract, and thus potentially reverse, neuropsychiatric outcomes. Indeed, knockdown of *RyR* genes in mouse brains elicited antidepressant-like effects [222]. Moreover, the prototypical mood stabilizer, lithium, has been suggested to exert some of its therapeutic effects by depleting free inositol, and the consequent attenuation of phosphoinositide signaling and IP_3_ production [223,224,225]. Diminished phosphoinositide signaling following lithium administration is thought to decrease IP_3_ production and thus, restrict IP_3_R-mediated ER Ca^2+^ release.

### 8.4. Peripheral Neuropathies

Charcot Marie Tooth disease (CMT) constitutes a spectrum of peripheral neuropathies associated with the degeneration of the long neuronal processes of motor and sensory neurons. Mutations in many genes are associated with CMT, and at least a subset of these lead to disease-causing defects in ER Ca^2+^ homeostasis and MAM function. A quintessential example illustrating the involvement of MAMs in this disease is CMT2A, which is caused by mutations in the gene encoding a mitochondrial protein, mitofusin-2 [226]. Further supporting a role for mitochondrial proteins in the pathophysiology of CMT, mutations or loss of the gene encoding GDAP1 lead to CMT2K and CMT4A [227]. However, in contrast to CMT2A, *GDAP1* mutations have been shown to result in decreased store-operated Ca^2+^ entry [227,228,229]. Forms of CMT attributed to mutations in the gene for peripheral myelin protein 22 (*PMP22*) also result in diminished store-operated Ca^2+^ entry, but in Schwann cells [230]. These data point to the complexities of ER-driven Ca^2+^ entry mechanisms in forms of CMT.

Although the exact role of mitofusin-2 in the regulation of Ca^2+^ homeostasis remains poorly understood, deletion or CMT2A-associated mutations in mitofusin-2 have been reported to prevent elevations in mitochondrial [Ca^2+^] in response to IP_3_R activation [226]. In contrast, another study has reported increased interorganellar Ca^2+^ transfer upon the knockdown of mitofusin-2 [231]. The conflicting findings regarding the role of mitofusin-2 likely reflect hitherto undescribed roles for additional factors that modulate ER–mitochondrial Ca^2+^ transfer. In addition, mitofusin-2 could be regulating attachment of a variety of different organelles, such as the overall effects of mitofusin-2 knockdown, which is highly context-dependent. Indeed, the association between the ER and the plasma membrane necessary for STIM-dependent activation of store-operated Ca^2+^ entry has also been proposed to involve mitofusin-2 [232]. 

### 8.5. Neurodegenerative Diseases Directly Attributed to ER Ca^2+^ Release Channels

Neurodegeneration refers to the progressive loss of neuronal function, often resulting from premature neuronal demise. In general, neurodegenerative diseases represent neurological diseases with the clearest and best-documented involvement of ER Ca^2+^ dyshomeostasis. This notion is exemplified by the findings that mutations in the gene encoding IP_3_R1, *ITPR1*, result in forms of spinocerebellar ataxia (SCA15 and SCA29) and Gillespie syndrome [233,234,235,236,237,238,239,240,241,242]. SCA15 is an adult-onset disease that presents with autosomal dominant cerebellar ataxia and attendant gait impairment due to heterozygous deletions spanning the *ITPR1* locus [233,234,235,236]. Mice that are heterozygous for a spontaneously arising *Itpr1* loss-of-function allele also exhibit severe cerebellar degeneration leading to abnormal locomotion starting around postnatal day 14 [234]. The exceptionally high abundance of IP_3_R1 in the cerebellar Purkinje neurons [234,243] could explain the haploinsufficiency observed upon deletion of one of the two copies of the gene encoding the channel. Constitutive homozygous deletion of *Iptr1* in mice leads to significant embryonic lethality [243]. Of the few animals that are born, all exhibit severe ataxia, tonic-clonic seizures, and die by the weaning period [243]. 

SCA29 is an infantile-onset form of the disease, and is associated with cerebellar atrophy, hypotonia from infancy, non-progressive ataxia, and related psychomotor deficiencies [238]. Patients with SCA29 are generally heterozygous for missense mutations in *ITPR1* that occur in exons encoding the IP_3_-biding domain of the protein, and thus, diminish or abolish channel function [237,238]. While most cases of SCA stem from decreased IP_3_R1 activity as the consequence of haploinsufficiency, one missense variant (IP_3_R1^R36C^) results in elevated IP_3_-binding affinity and sustained channel activation [244]. Interestingly, these patients also present with typical features of SCA, including gait abnormalities and delayed motor development [244]. These data point to the importance of *ITPR1* dosage and maintenance of channel activity within a ‘Goldilocks’ zone for Purkinje neuron viability.

Gillespie syndrome, which is a rare congenital disorder characterized by hypotonia, ataxia due to progressive cerebellar atrophy, and intellectual disability, is also caused by autosomal recessive or dominant missense mutations in *ITPR1* [240,241,242]. *ITPR1* mutations in Gillespie syndrome are varied, and often involve partial expression of the wild-type gene accompanied by truncated variants that exert dominant-negative effects [240,241,242]. Although the cerebellar and motor outcomes in Gillespie syndrome mimic those in SCA, an intriguing feature that distinguishes Gillespie syndrome is the appearance of partial aniridia [240,241,242,245]. The mechanisms underlying the specificity of aniridia in Gillespie syndrome remain unknown, and mice with *ITPR1* mutations do not exhibit aniridia [242]. 

### 8.6. Age-Related Neurodegenerative Diseases

**Figure 6:**
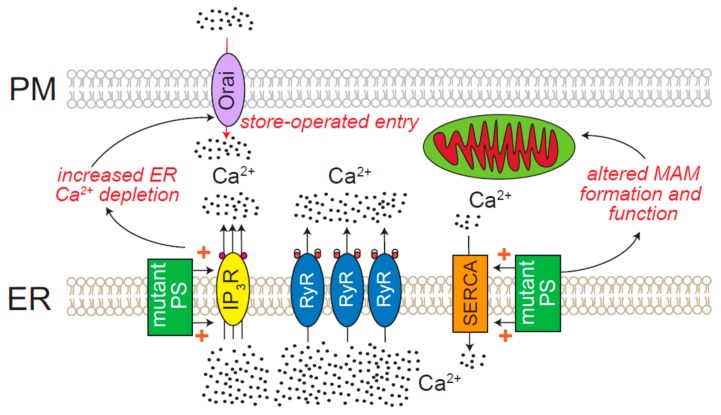
Mechanisms of ER Ca^2+^ dyshomeostasis in Alzheimer’s disease (AD). Mutated presenilin (PS) increases ER Ca^2+^ release. Various models of AD display increased expression of *RyR*, which can also produce elevated Ca^2+^ release from the ER. Additionally, mutated PS stimulates sarco/endoplasmic reticulum Ca^2+^-ATPase (SERCA), which can fuel further release through ER Ca^2+^ channels. Increased ER Ca^2+^ store depletion can activate store operated Ca^2+^ entry. Function of mitochondria associated membranes (MAMs) are also altered by mutated PS.

Age-related neurodegenerative diseases (Alzheimer’s disease (AD), Parkinson’s disease (PD), Huntington’s disease (HD), and amyotrophic lateral sclerosis (ALS)) exhibit ER Ca^2+^ dyshomeostasis. A “Ca^2+^ hypothesis” has been advanced to bridge the mechanistic gap between amyloid accumulation and cognitive decline in AD [246]. This model is built on the understanding that AD neurons exhibit elevated cytosolic Ca^2+^ levels. For instance, baseline cytosolic [Ca^2+^] in neurons from the 3xTg-AD mouse model is double that in control cells [247]. Mutated variants of presenilin, which are a familial cause of AD, potentiate the activity of IP_3_Rs via direct regulation of channel gating or other indirect mechanisms (Figure 6) [65,248,249,250]. A number of other studies have also reported increases in the abundance and activity of RyRs in AD mouse models, which has raised the possibility of treating AD with the RyR antagonist, dantrolene [59,251,252]. Adding to this complexity, presenilins also regulate the activity of SERCA, ER–mitochondrial transfer of Ca^2+^ and phospholipids, mitochondrial ATP production, and overall cellular Ca^2+^ homeostasis (Figure 6) [253,254,255]. In contrast, amyloid β oligomers have been shown to increase the transfer of Ca^2+^ from the ER to mitochondria, leading to mitochondrial Ca^2+^ overload, sustained store depletion, and toxic activation of store-operated Ca^2+^ entry [256]. As mentioned in a previous section, ER Ca^2+^ release and store-operated Ca^2+^ entry (Figure 6) can lead to calcineurin-dependent nuclear translocation of NFAT, and potentiate cell death in mature neurons [98,102,103]. Pointing to the involvement of this axis in AD, inhibition of the calcineurin/NFAT pathway alleviates amyloid β-induced neurodegeneration in a murine model of the disease [257]. An additional consequence of ER Ca^2+^ store depletion is ER stress and activation of the unfolded protein response (UPR), which also contributes to AD pathology [258,259,260]. Despite these studies pointing to a pathological role for excessive ER Ca^2+^ release in AD, further studies are needed to reconcile the conflicting reports suggesting either absent or opposite changes in ER Ca^2+^ homeostasis in AD [261,262,263,264].

One view of the pathological mechanisms underlying PD, a disease associated with the loss of dopaminergic neurons of the substansia nigra, revolves around the concept of dysregulated MAMs and perturbations in ER–mitochondria Ca^2+^ signaling [265]. In induced pluripotent stem cell (iPSC)-derived dopaminergic neurons from familial PD patients with triplication of the α-synuclein gene, interactions between the ER and mitochondrial counterparts of MAMs VAPB and PTPIP51, respectively, are disrupted [266]. The resulting cessation of interorganellar Ca^2+^ transfer decouples IP_3_R activation from mitochondrial Ca^2+^ elevation, and thus, limits mitochondrial ATP production. Deprivation of ATP in the dopaminergic neurons of the substansia nigra may be especially consequential, as these cells perform a particularly energy demanding pacemaker function that requires tight homeostatic control over ionic gradients [265]. As is the case in AD, calcineurin and NFAT mediate α-synuclein-induced loss of dopaminergic neurons [267,268]. Since ER Ca^2+^ release and store-operated entry activate calcineurin/NFAT-dependent neurotoxicity, these findings are consistent with augmented ER Ca^2+^ release as being a causal insult in PD.

HD is a neurodegenerative disease caused by CAG repeat extensions in the gene encoding Huntingtin, and is characterized by extensive loss of neurons in the striatum resulting in characteristic chorea and progressive dementia [269]. IP_3_R1–GRP78 coupling and enhanced transfer of Ca^2+^ between the ER and mitochondria have been suggested to underlie ER stress and degeneration in a mouse model of HD [270]. In agreement with these reports, expression of mutant Huntingtin (i.e., with polyglutamine repeat expansions), but not wild type Huntingtin, in murine medial spiny neurons enhanced IP_3_-mediated Ca^2+^ release [271]. Genetic studies in *Drosophila* further support this interaction given that knockdown of IP_3_R suppressed neurodegeneration in a fly HD model [272]. As expected from elevated ER Ca^2+^ release, store-operated Ca^2+^ entry is enhanced in striatal neurons of a mouse model of HD, which underlies the synaptic loss observed in those animals [273]. A unique feature of HD in the context of ER Ca^2+^ is that mutant Huntingtin exists in a complex with IP_3_R1 via interaction with the C-terminus of the channel [274]. Thus, expression of a C-terminal fragment of IP_3_R1 attenuates the interaction between mutant Huntingtin and IP_3_R1 leading to the mitigation of neuronal loss in a mouse model of the disease [274].

**Figure 7:**
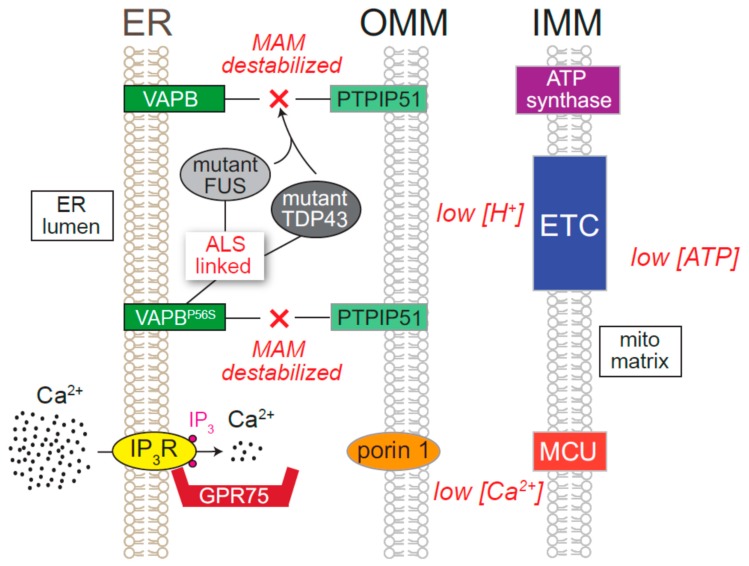
Mechanisms of mitochondria-associated membrane (MAM) dysfunction in amyotrophic lateral sclerosis (ALS). Various ALS-associated mutations lead to breakdown of MAMs. Mutated VAPB (VAPB^P56S^) leads to decreased binding with PTPIP51 and mutant FUS and TDP-43 disrupt the VAPB-PTPIP51 interaction, diminishing Ca^2+^ transfer from the ER to the mitochondria. Lower Ca^2+^ in the mitochondrial matrix results in less oxidative phosphorylation and reduced ATP production.

Various lines of evidence also point to ER Ca^2+^ dyshomeostasis stemming from perturbations in ER Ca^2+^ release and MAM assembly as being important pathological mechanisms in ALS, a lethal neurodegenerative disease characterized by motor neuron degeneration [275]. A familial form of ALS (ALS8) is caused by a mutations in the MAM resident protein, VAPB (for example, VAPB^P56S^), which participates in ER-mitochondrial tethering [275,276,277]. Although ALS8 is a rare form of ALS, decreased VAPB protein abundance has been observed in spinal cord motor neurons from patients with sporadic ALS [278]. In other forms of familial ALS, such as those caused by mutated variants of FUS and TDP-43, VAPB–PTPIP51 interactions are comprised with attendant decreases in mitochondrial [Ca^2+^] and ATP production (Figure 7) [279,280]. In accordance with the relationship between TDP-43 and ER Ca^2+^ homeostasis, a worm model of ALS exhibits ER Ca^2+^ dyshomeostasis that led to decline of neuronal function [281]. ALS-causing mutations in the gene encoding Sigma receptor-1, and C9orf72 hexanucleotide expansions (a leading cause of both sporadic and familial forms of ALS in populations with European ancestry) are also associated with ER Ca^2+^ dyshomeostasis due to combinations of elevated IP_3_R activity and diminished uptake into stores [282,283]. 

## 9. Closing Remarks

In this review, we have described the involvement of ER Ca^2+^ homeostasis in neuronal functions ranging from development and plasticity to gene expression and cell survival. We also provided examples of neurological and neurodegenerative diseases that arise when the processes regulating the movement of Ca^2+^ in and out of the ER go awry. It is important to note that despite being one of the most extensively studied organelles, much still remains to be known about ER Ca^2+^ and its relationship to neuronal function and disease. In particular, we anticipate a proliferation of studies focused on the domains that the ER forms with other organelles such as the mitochondria, endolysosomes, and the plasma membrane. These studies should go a long way to illustrate that in the ultimate analysis, intracellular organelles function in an ecosystem that requires invaluable contributions from all the players. By remaining open to these possibilities, we can hope for novel therapies for a variety of debilitating neurological diseases that arise from miscommunication between neuronal organelles.
